# The Change in Environmental Variables Linked to Climate Change Has a Stronger Effect on Aboveground Net Primary Productivity Than Does Phenological Change in Alpine Grasslands

**DOI:** 10.3389/fpls.2021.798633

**Published:** 2022-01-04

**Authors:** Jiangwei Wang, Meng Li, Chengqun Yu, Gang Fu

**Affiliations:** ^1^Lhasa Plateau Ecosystem Research Station, Key Laboratory of Ecosystem Network Observation and Modeling, Institute of Geographic Sciences and Natural Resources Research, Chinese Academy of Sciences, Beijing, China; ^2^University of Chinese Academy of Sciences, Beijing, China; ^3^School of Geographic Sciences, Nantong University, Nantong, China

**Keywords:** green-up date, growing season length, warming, precipitation, alpine ecosystem, Tibetan Plateau

## Abstract

More and more studies have focused on responses of ecosystem carbon cycling to climate change and phenological change, and aboveground net primary productivity (ANPP) is a primary component of global carbon cycling. However, it remains unclear whether the climate change or the phenological change has stronger effects on ANPP. In this study, we compared the effects of phenological change and climate change on ANPP during 2000–2013 across 36 alpine grassland sites on the Tibetan Plateau. Our results indicated that ANPP showed a positive relationship with plant phenology such as prolonged length of growing season and advanced start of growing season, and environmental variables such as growing season precipitation (GSP), actual vapor pressure (E_a_), relative humidity (RH), and the ratio of GSP to ≥5°C accumulated temperature (GSP/AccT), respectively. The linear change trend of ANPP increased with that of GSP, E_a_, RH, and GSP/AccT rather than phenology variables. Interestingly, GSP had the closer correlation with ANPP and meanwhile the linear slope of GSP had the closer correlation with that of ANPP among all the concerned variables. Therefore, climate change, mainly attributed to precipitation change, had a stronger effect on ANPP than did phenological change in alpine grasslands on the Tibetan Plateau.

## Introduction

Aboveground net primary productivity (ANPP) is a primary component of global carbon cycling in terrestrial ecosystems and understanding its drivers has long been a goal of ecology (Wu et al., 2011; Robinson et al., 2013). Climate change, especially warming and precipitation change, is a vital abiotic variable in driving ANPP variations (Rustad et al., 2001; Wu et al., 2011). More and more studies have analyzed responses of ANPP to warming and precipitation variations (Klein et al., 2007; Wu et al., 2011; Wang et al., 2012; Fu Z. et al., 2015). Warming and water availability not only directly affect plant physiology related to plant photosynthesis and in turn plant photosynthesis (Fu G. et al., 2015) but also indirectly affect plant growth by altering nitrogen availability, species composition, and plant phenology (Wan et al., 2005; Wang et al., 2012). The net effect of climate change on ANPP is dependent on the relative strength of climate change-induced direct effect and indirect effect on ANPP. Many studies have indicated that the effect of precipitation change on ANPP is stronger than that of warming (Wu et al., 2011; Shen et al., 2014), while some other studies have found quite the contrary results (Wang et al., 2012). Therefore, it remains unclear on the relative effects of warming and precipitation change on ANPP.

Plant phenology (e.g., start of growing season, SGS; end of growing season, EGS; and length of growing season, LGS), as a critical aspect of biological systems (Dorji et al., 2013), is an important biotic variable in affecting ANPP (Berdanier and Klein, 2011). A growing number of studies have focused on the correlations between plant phenology and plant productivity (Parmentier et al., 2011; Miller and Smith, 2012; Kross et al., 2014), while there are no consistent findings, with positive (Wu et al., 2012), negative (Jia et al., 2010), or no effects (Parmentier et al., 2011; Zhu et al., 2017) of prolonged LGS on plant productivity. Plant phenology itself is sensitive to climate change, and both warming and water availability can alter plant phenology (Prieto et al., 2009; Chen et al., 2011; Shen et al., 2011; Westergaard-Nielsen et al., 2017). These diverse findings imply that the effects of plant phenology on plant productivity can be regulated by climate changes (Wu et al., 2012; Wang et al., 2017). Moreover, these previous studies have mainly focused on gross primary production, net primary production, and net ecosystem production (Piao et al., 2007; Zhao and Liu, 2012; Takagi et al., 2015) rather than ANPP (Baptist et al., 2010; Berdanier and Klein, 2011). Therefore, the effects of plant phenology on ANPP remains unclear.

The Tibetan Plateau is one of the most sensitive regions to climate change and is mainly covered by alpine grasslands. A large number of studies have examined plant phenological changes and their driving mechanisms related to climate change in alpine grasslands on the Tibetan Plateau (Piao et al., 2011; Shen, 2011; Cong et al., 2012; Dorji et al., 2013; Ding et al., 2016; Ganjurjav et al., 2016b). However, only a few studies have compared the effects of climate change and phenological change on productivity (i.e., gross primary productivity, net primary productivity, and net ecosystem productivity) in alpine grasslands on the Tibetan Plateau (Yang et al., 2015; Wang et al., 2017; Zhu et al., 2017), and no studies have investigated the responses of ANPP to climate change and phenological change. Alpine grasslands are main pasture, and the ANPP in alpine grasslands plays vital roles in sustainable development of pastoral livestock industry on the Tibetan Plateau. Therefore, in this study, we analyzed the correlations of ANPP with phenological variables (i.e., SGS, EGS, and LGS) and climate variables (e.g., precipitation and temperature). The main objective of this study was to better predict future changes in ANPP under global change by comparing the relative effects of climate and phenological variables on ANPP in alpine grasslands on the Tibetan Plateau.

## Materials and Methods

### Aboveground Biomass Sampling and Aboveground Net Primary Productivity Estimation

Articles published in 2000–2015 were searched using the Web of Science and the China National Knowledge Infrastructure to obtain aboveground biomass (AGB) in alpine grasslands on the Tibetan Plateau. There were 195 AGB data (2.99–759.19 g m^–2^), which were sampled during July–August of 2000–2013. There were 123 sampling sites (Supplementary Figure 1). Moderate Resolution Imaging Spectroradiometer (MODIS) NDVI data (MOD13A3, Collection 6) during June–September of 2000–2013 were downloaded. The relationship between AGB and NDVI was developed (Supplementary Figure 2). Then, the AGB were obtained during June–September of 2000–2013 in alpine grasslands on the whole Tibetan Plateau using the models mentioned above. Many previous studies, which were conducted in alpine grasslands on the Tibetan Plateau, have indicated that the maximum AGB during the growing season could be treated as aboveground net primary production (ANPP; Klein et al., 2007; Wang et al., 2012). Therefore, the maximum AGB during June–September was treated as ANPP in this study.

### MOD13A2 and Phenological Metrics

NDVI data were obtained from MODIS vegetation indices product (MOD13A2, Collection 6). The spatial and temporal resolutions of MOD13A2 NDVI are 1 km × 1 km and 16 days, respectively. Images collected during 2000–2013 were used for this study. The Timesat-SG method was used to estimate SGS, EGS, and LGS (Cong et al., 2012). In this study, 20 and 50% was used as the two dynamic thresholds to determine SGS and EGS, respectively (Cong et al., 2012; Wang et al., 2017).

### Climate Data

**FIGURE 1:**
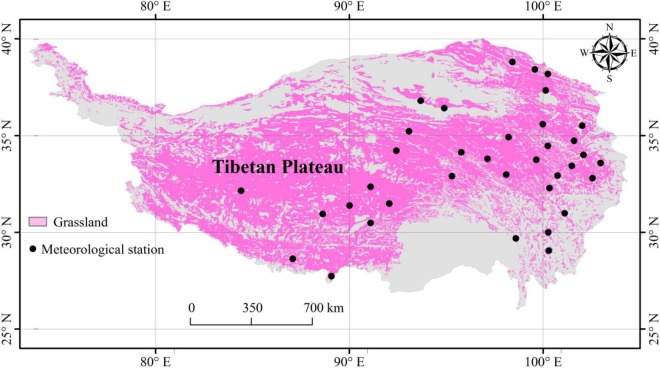
Location of 36 meteorological stations on the Tibetan Plateau.

Climate data were obtained from 36 meteorological stations (Figure 1) of the China Meteorological Data Sharing Service System (Deng et al., 2013; Zhang et al., 2013). The climate data included growing-season precipitation (GSP), actual vapor pressure (E_a_), relative humidity (RH), minimum relative humidity (RH_min_), vapor pressure deficit (VPD), air temperature (T_a_), minimum air temperature (T_amin_), maximum air temperature (T_amax_), ≥5°C accumulated temperature (AccT), and the ratio of GSP to AccT (GSP/AccT). The GSP/AccT ratio is a synthesized factor of temperature and precipitation, which has been used in several previous studies (Wang et al., 2013; Wu et al., 2014; Fu et al., 2018). The GSP, E_a_, RH, RH_min_, and VPD could be used as variables related to water availability, and T_a_, T_amin_, T_amax_, and AccT could be used as variables related to temperature.

### Statistical Analysis

Although spatial interpolation is a general approach to obtain climate data at regional scale (Attorre et al., 2007; Hashimoto et al., 2008), there remains some errors in the interpolated climate data, especially for precipitation (Hashimoto et al., 2008; Fu et al., 2017). Therefore, we only analyzed the effects of climate change on aboveground productivity at the 36 meteorological stations in this study. Simple linear regressions of ANPP with SGS, EGS, LGS, GSP, E_a_, RH, RH_min_, VPD, T_a_, T_amin_, T_amax_, AccT, and GSP/AccT were performed using all the data, respectively. Correlation coefficients of ANPP with SGS, EGS, LGS, GSP, Ea, RH, RH_min_, VPD, T_a_, T_amin_, T_amax_, AccT, and GSP/AccT were conducted for each site, respectively. The largest absolute value of correlation coefficients of ANPP with these concerned variables was treated as the dominated factor. The variation of ANPP was partitioned using climate variables (i.e., GSP, Ea, RH, RH_min_, VPD, T_a_, T_amin_, T_amax_, AccT, and GSP/AccT) and phenological variables (i.e., SGS, EGS, and LGS) was partitioned using varpart function. Linear regression coefficients (i.e., slope) between ANPP, SGS, EGS, LGS, GSP, Ea, RH, RH_min_, VPD, T_a_, T_amin_, T_amax_, AccT, GSP/AccT, and time series (i.e., from 2000 to 2013) were conducted to obtain the temporal changes of these concerned parameters during 2000–2013 for each one of the 36 sites. The linear changes of ANPP, SGS, EGS, LGS, GSP, Ea, RH, RH_min_, VPD, T_a_, T_amin_, T_amax_, AccT, and GSP/AccT were labeled as slope_ANPP, slope_SGS, slope_EGS, slope_LGS, slope_GSP, slope_Ea, slope_RH, slope_RH_min_, slope_VPD, slope_T_a_, slope_T_amin_, slope_T_amax_, slope_AccT, and slope_GSP/AccT, respectively. Simple linear regressions of slope_ANPP with slope_SGS, slope_EGS, slope_LGS, slope_GSP, slope_Ea, slope_RH, slope_RH_min_, slope_VPD, slope_T_a_, slope_T_amin_, slope_T_amax_, slope_AccT, and slope_GSP/AccT were performed, respectively. The variation of slope_ANPP was partitioned using climate change (i.e., slope_GSP, slope_Ea, slope_RH, slope_RH_min_, slope_VPD, slope_T_a_, slope_T_amin_, slope_T_amax_, slope_AccT, and slope_GSP/AccT) and phenological change (i.e., slope_SGS, slope_EGS, and slope_LGS) was partitioned using varpart function.

## Results

### Climate Change

The change trends of climate variables are listed in Supplementary Table 1. The GSP in seven sites showed decreasing trends by −15.23 to −0.14 mm a^–1^, while that in the other 29 sites showed increasing trends by 0.90–13.27 mm a^–1^. The E_a_ in 20 sites showed decreasing trends by −0.02 to −0.12 kPa a^–1^, while that in the other 16 sites showed increasing trends by 0.001–0.04 kPa a^–1^. The RH in 35 sites showed decreasing trends by −1.00 to −0.02% a^–1^, while that in the other one site showed an increasing trend by 0.07% a^–1^. The RH_min_ in all the 36 sites showed decreasing trends by −1.98 to −0.12% a^–1^. The VPD in only two sites showed decreasing trends by −0.04 to −0.002 kPa a^–1^, while that in the other 34 sites showed increasing trends by 0.002–0.13 kPa a^–1^. The T_a_ in seven sites showed decreasing trends by −0.07 to −0.01°C a^–1^, while that in the other 29 sites showed increasing trends by 0.02–0.11°C a^–1^. The T_amax_ in seven sites showed decreasing trends by −0.09 to −0.01°C a^–1^, while that in the other 29 sites showed increasing trends by 0.00–0.18°C a^–1^. The T_amin_ in seven sites showed decreasing trends by −0.08 to −0.01°C a^–1^, while that in the other 29 sites showed increasing trends by 0.00–0.15°C a^–1^. The AccT in only two sites showed decreasing trends by −5.60 to −5.28°C a^–1^, while that in the other 34 sites showed increasing trends by 2.02–45.48°C a^–1^. The GSP/AccT ratio in 14 sites showed decreasing trends by −0.01 to −0.0002 mm °C^–1^ a^–1^, while that in the other 22 sites showed increasing trends by 0.0001–0.02 mm °C^–1^ a^–1^.

### Phenological Change and Aboveground Net Primary Productivity Change

The change trends of phenology variables and ANPP are listed in Supplementary Table 1. The SGS in 25 sites showed decreasing trends by −2.53 to −0.05 day a^–1^, while that in the other 11 sites showed increasing trends by 0.10–1.54 day a^–1^. The EGS in 16 sites showed decreasing trends by −1.83 to −0.07 day a^–1^, while that in the other 20 sites showed increasing trends by 0.03–1.62 day a^–1^. The LGS in 12 sites showed decreasing trends by −2.23 to −0.06 day a^–1^, while that in the other 24 sites showed increasing trends by 0.002–3.89 day a^–1^. The decreases in SGS and, meanwhile, the increases in EGS resulted in the increases in LGS in 13 sites. The decreased magnitudes of SGS were greater than those of EGS, which caused the increases in LGS in the other eight sites. The increased magnitudes of EGS were greater than those of SGS, which resulted in the increases in LGS in the other three sites. The increases in SGS and, meanwhile, the decreases in EGS caused the decreases in LGS in four sites. The increased magnitudes of SGS were greater than those of EGS, which resulted in the decreases in LGS in the other four sites. The decreased magnitudes of EGS were greater than those of SGS, which caused the decreases in LGS in the other four sites.

The ANPP in 14 sites showed decreasing trends by −10.48 to −0.03 g m^–2^ a^–1^, while that in the other 22 sites showed increasing trends by 0.01–8.62 g m^–2^ a^–1^.

### Effects of Climate Change and Phenological Change on Aboveground Net Primary Productivity

**FIGURE 2:**
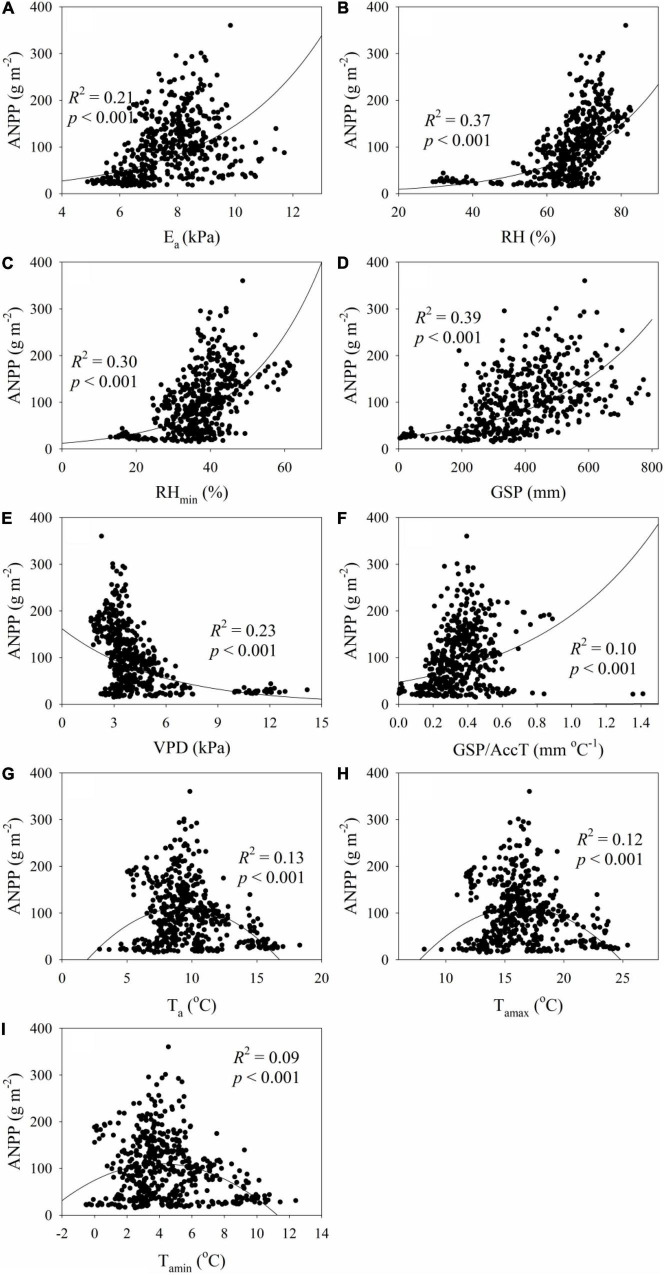
Relationships **(A)** between aboveground net primary production (ANPP) and growing season average actual vapor pressure (E_a_), **(B)** between ANPP and growing season average relative humidity (RH), **(C)** between ANPP and growing season minimum RH (RH_min_), **(D)** between ANPP and growing season total precipitation (GSP), **(E)** between ANPP and growing season average vapor pressure deficit (VPD), **(F)** between ANPP and the ratio of GSP to accumulated temperature (GSP/AccT), **(G)** between ANPP and growing season average air temperature (T_a_), **(H)** between ANPP and maximum air temperature (T_amax_), and **(I)** between ANPP and minimum air temperature (T_amin_).

**FIGURE 3:**
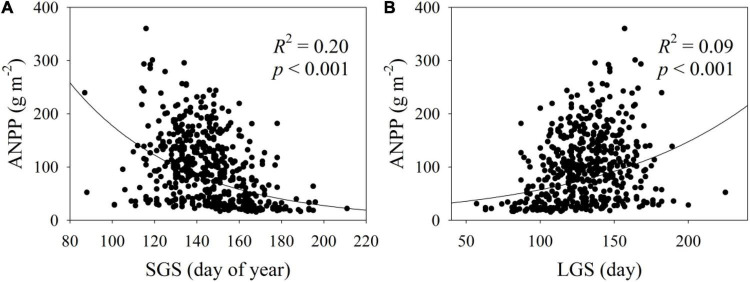
Relationships **(A)** between ANPP and start of growing season (SGS) and **(B)** between ANPP and length of growing season (LGS).

**FIGURE 4:**
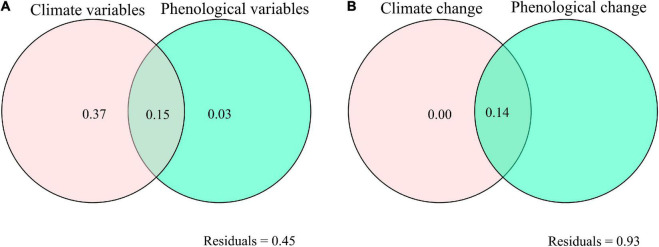
Varpart analysis, showing **(A)** the exclusive and shared effects of climate variables [growing season precipitation (GSP), E_a_, RH, minimum RH, vapor pressure deficit, air temperature, minimum air temperature, maximum air temperature, accumulated ≥5°C air temperature, ratio of GSP to accumulated ≥5 air temperature], and phenological variables [LGS, SGS, and end of growing season (EGS)] on ANPP, **(B)** the exclusive and shared effects of climate change (linear slopes of GSP, E_a_, RH, minimum RH, vapor pressure deficit, air temperature, minimum air temperature, maximum air temperature, accumulated ≥5°C air temperature, ratio of GSP to accumulated ≥5°C air temperature), and phenological change (linear slopes of LGS, SGS, and EGS) on the changes in ANPP.

The ANPP increased exponentially with E_a_, RH, RH_min_, GSP, GSP/AccT and LGS, but decreased exponentially with VPD and SGS (Figures 2, 3). The ANPP showed a significant quadratic correlation with T_a_, T_amax_, and T_amin_ (Figure 2). The E_a_, RH, RH_min_, GSP, VPD, GSP/AccT, T_a_, T_amax_, T_amin_, SGS, and LGS explained significantly 21, 37, 30, 39, 23, 10, 13, 12, 9, 20, and 9% variation of ANPP, respectively (Figures 2, 3). Moreover, the correlation coefficients of ANPP with AccT (*p* = 0.181) and EGS (*p* = 0.068) were not significant. The varpart analysis showed that climate variables and phenological variables exclusivity explained 37 and 3% variation of ANPP, respectively, and they together explained 15% variation of ANPP (Figure 4A); i.e., the variations of ANPP were more explained by climate variables rather than phenology variables.

**FIGURE 5:**
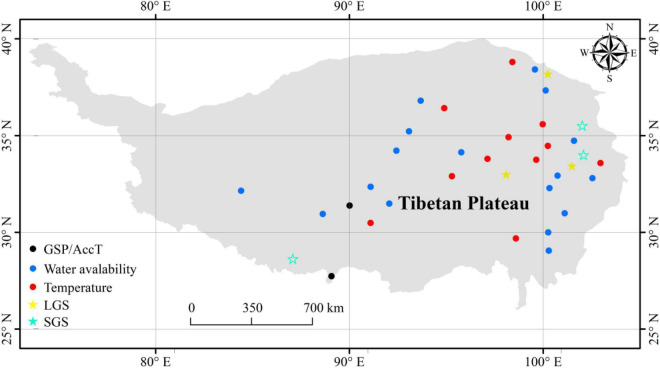
The spatial distribution of the dominant variables of ANPP.

**FIGURE 6:**
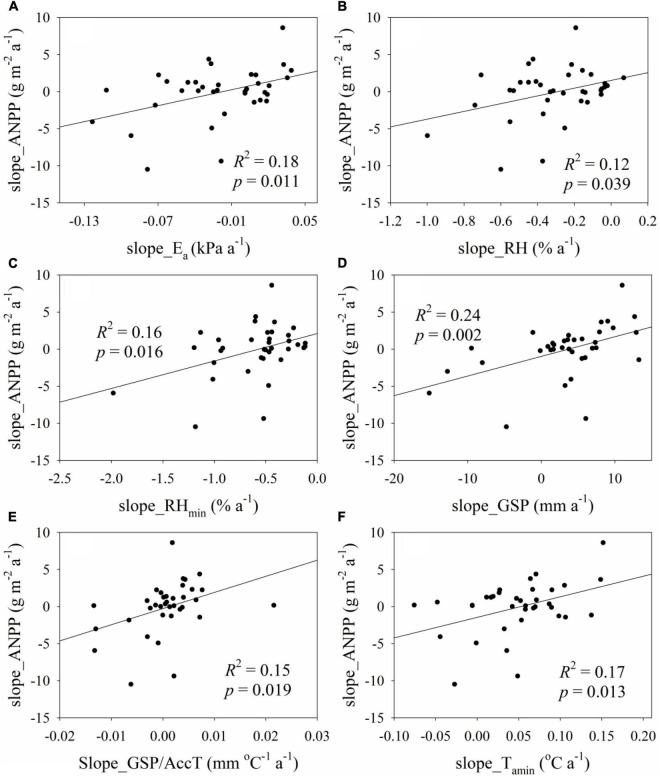
Relationships **(A)** between linear slope of ANPP (slope_ANPP) and that of growing season average E_a_ (slope_E_a_), **(B)** between slope_ANPP and that of growing season average RH (slope_RH), **(C)** between slope_ANPP and that of growing season minimum RH (slope_RH_min_), **(D)** between slope_ANPP and that of growing season average precipitation (slope_GSP), **(E)** between slope_ANPP and that of the ratio of GSP to accumulated temperature (slope_GSP/AccT), and **(F)** between slope_ANPP and that of minimum air temperature (slope_T_amin_).

The changes of ANPP were dominated by SGS at three sites, by LGS at three sites, by water availability at 17 sites, by temperature variables at 11 sites, and by GSP/AccT ratio at two sites (Figure 5); i.e., phenology changes predominated ANPP changes at only six sites, while climate changes predominated ANPP changes at 30 sites. The slope_ANPP increased significantly with increasing slope_E_a_, slope_RH, slope_RH_min_, slope_GSP, slope_GSP/AccT, and slope_T_amin_ (Figure 6). However, slope_ANPP was not linearly correlated with slope_VPD (*p* = 0.141), slope_T_a_ (*p* = 0.262), slope_AccT (*p* = 0.709), slope_SGS (*p* = 0.213), slope_EGS (*p* = 0.106), slope_LGS (*p* = 0.940), and slope_T_amax_ (*p* = 0.622). Climate change exclusively explained about 0.4% variation of slope_ANPP, but phenology change did not exclusively explain the variation of slope_ANPP (Figure 4B).

## Discussion

### Effects of Climate Change on Aboveground Net Primary Productivity

There was the most likely optimum temperature for ANPP in alpine grasslands (Figure 2), which was in line with several previous studies (Wang et al., 2013). This finding implied that climate warming may not always increase ANPP in alpine regions. In fact, there were diverse responses of ANPP to climate warming in alpine regions, with increases (Wang et al., 2012; Ganjurjav et al., 2015), decreases (Klein et al., 2007; Ganjurjav et al., 2016a), or no change (Natali et al., 2012). These diverse effects of climate warming on ANPP were attributed to the following mechanisms. First, ANPP was more sensitive to warming in the colder environment in alpine regions (Rustad et al., 2001). Second, Shi et al. (2014) ascribed the relatively low effect of warming on ANPP to the that alpine plants had a low optimal temperature, high-temperature adaptation, and strong resilience to short-term temperature fluctuations. Third, warming generally resulted in soil drying (Lu et al., 2013), and the lower limit of soil moisture was 11.8% for alpine grassland growth (Ma et al., 2004). Warming-induced soil drying can dampen the effect of increased temperature on ANPP by reducing leaf area and inducing stomatal closure (Broeckx et al., 2014). Fourth, warming-induced decreases in species richness and diversity can influence warming effects on ANPP because ANPP had some relations with species diversity (Ma et al., 2010; Wang et al., 2013; Wu et al., 2014). Fifth, warming can accelerate plant maturity and actually shorten LGS (Li et al., 2004). The diverse correlations between plant productivity and LGS in this study could result in inconsistent responses of ANPP to warming.

Precipitation has increased by 0.67 mm a^–1^ during 1961–2010 on the Tibetan Plateau (Li X. Y. et al., 2016) and will continue to increase in the 21st century (Ji and Kang, 2013). Increased precipitation will most likely increase ANPP in alpine grasslands on the Tibetan Plateau (Figure 2). Likewise, ANPP increased significantly with increasing GSP in alpine grasslands on the Northern Tibetan Plateau (Wu et al., 2014). ANPP increased with increasing GSP across the widely distributed temperate and alpine grasslands of China (Ma et al., 2010). A previous meta-analysis found that increased precipitation increased ANPP, while decreased precipitation reduced ANPP at a global scale (Wu et al., 2011).

Water availability had stronger effects on ANPP than did temperature (Figures 2, 6), which was consistent with several previous studies (Wu et al., 2011; Fu and Shen, 2016a; Xu et al., 2016). For example, rainfall fluctuation had a more profound effect on the ANPP dynamics than temperature variation in the Tibetan alpine grasslands (Shi et al., 2014). Growing-season maximum normalized difference vegetation index had a closer correlation with water availability than temperature across the Tibetan Plateau during 2000–2012 (Shen et al., 2014).

### Effects of Phenological Changes on Aboveground Net Primary Productivity

The effect of SGS on ANPP was stronger than that of EGS and LGS across all the years and sites (Figure 3). Likewise, the effect of SGS on annual net ecosystem production was stronger than that of LGS in alpine shrubland on the Qinghai-Tibetan Plateau (Li H. Q. et al., 2016). SGS had a higher correlation with net primary production than did EGS in alpine ecosystems on the Tibetan Plateau (Yang et al., 2015; Wang et al., 2017). SGS had a greater correlation with gross primary production than did EGS in temperate deciduous broadleaved forests in North America (Zhao and Liu, 2012).

Across all the years and sites, ANPP increased with prolonged SGS and LGS (Figure 3). Likewise, ANPP increased with LGS across a suite of high elevation meadows in the United States and Asia (Berdanier and Klein, 2011). Both advanced SGS and prolonged LGS showed a positive effect on gross primary production across eight larch forests in East Asia (Takagi et al., 2015). In the Northern Hemisphere, LGS was strongly correlated with gross primary production and net primary production (Piao et al., 2007). However, there were spatial variations in SGS, EGS, and LGS changes during the past 14 years, which could also be observed by several previous studies (Piao et al., 2007; Shen et al., 2011; Song et al., 2011). The spatial variations in phenological changes, in turn, may partially result in diverse relationships between ANPP and these three phenological variables among all the 36 sites. Previous studies also found that responses of plant productivity to LGS were dependent on sites (Berdanier and Klein, 2011). For example, a prolonged LGS increased aboveground biomass under warming conditions in a tallgrass prairie, United States (Wan et al., 2005). There was no increase in alpine snowbed ANPP in response to experimental prolonged LGS (Baptist et al., 2010). Although experimental warming extended LGS, there was no increase in gross primary production and species-level coverage in response to experimental warming in a semiarid grassland (Xia and Wan, 2012). In addition, both extended LGS and advanced SGS resulted in less net ecosystem productivity in a subalpine forest in the Colorado Rocky Mountains (Jia et al., 2010).

These diverse responses of plant productivity to plant phenological change could be contributed to the following mechanisms. First, prolonged LGS, advanced SGS, and delayed EGS may lead to a greater temporal species overlap, reduce phenological complementarity, and increase water and nutrient competition among species (Xia and Wan, 2012; Dorji et al., 2013). This may cause species loss, which, in turn, increased complexity on the relationships between plant productivity and plant phenology, considering the diverse correlations between plant productivity and species diversity in alpine regions (Wang et al., 2012, 2013; Wu et al., 2014). Second, plant intrinsic developmental capacities (e.g., periodic species and aperiodic species) varied among species (Baptist et al., 2010). Third, advanced SGS may lead to potential detrimental effects of early frosts, which, in turn, result in a reduction in plant productivity (Baptist et al., 2010). Fourth, plant photosynthetic CO_2_ uptake depended on snowmelt (Jia et al., 2010). Advanced SGS may result in earlier snowmelt, which, in turn, affected plant productivity (Jia et al., 2010). Fifth, SGS showed a negative correlation with GSP and E_a_, and LGS showed a positive correlation with GSP and E_a_ in this study. This indicated that advanced SGS and prolonged LGS can increase water availability during the growing season. Accumulated precipitation within phenological duration was positively related to plant productivity (Xia and Wan, 2012). Sixth, plant growth in alpine regions is generally nitrogen-limited (Fu and Shen, 2016b). A delayed SGS and shortened LGS had a greater positive effect on ANPP by increasing soil fertility than did an advanced SGS and prolonged LGS (Baptist et al., 2010).

### Stronger Effect of Climate Change on Aboveground Net Primary Productivity Than That of Phenological Change

Our findings implied that climate variables (especially GSP) rather than phenological variables (i.e., LGS and SGS) predominated ANPP changes across all the alpine grassland sites on the Tibetan Plateau. These results were in line with previous studies on the response of plant productivity to climate and phenological changes. For example, shortened growing seasons did not affect both gross primary production and net primary production under experimental warming, which was attributed to the seasonal variation of precipitation in an alpine meadow on the Tibetan Plateau (Zhu et al., 2017). The changes in gross primary production and species-level coverage were positively correlated to the accumulated precipitation within phenological duration but not the length of phenological duration in a semiarid grassland (Xia and Wan, 2012). Gross primary production did not increase with longer LGS, but increased with growing-season temperature sum in the northeastern Siberian tundra (Parmentier et al., 2011).

## Conclusion

In this study, we compared the effect of phenological variables and climate variables on aboveground net primary production in alpine grasslands on the Tibetan Plateau. LGS and SGS explained 9 and 20% variation of ANPP, while GSP, RH, E_a_, VPD, and GSP/AccT explained 39, 37, 21, 23, and 10% variation of ANPP, respectively. Moreover, the linear slope of ANPP showed a positive relationship with that of GSP (*R*^2^ = 0.24), RH (*R*^2^ = 0.12), E_a_ (*R*^2^ = 0.18), and GSP/AccT (*R*^2^ = 0.15) but did not correlate to that of LGS and SGS. Therefore, precipitation change predominated the variation of ANPP and responses of ANPP to climate change were greater than those of phenological change.

## Data Availability Statement

The datasets presented in this article are not readily available because the datasets generated for this study are available on request to the corresponding author. Requests to access the datasets should be directed to GF.

## Author Contributions

JW wrote the first manuscript, which revised by GF and CY. ML collected the data. All authors contributed to the article and approved the submitted version.

## Conflict of Interest

The authors declare that the research was conducted in the absence of any commercial or financial relationships that could be construed as a potential conflict of interest.

## Publisher’s Note

All claims expressed in this article are solely those of the authors and do not necessarily represent those of their affiliated organizations, or those of the publisher, the editors and the reviewers. Any product that may be evaluated in this article, or claim that may be made by its manufacturer, is not guaranteed or endorsed by the publisher.
