# Supplementary material for: The Change in Environmental Variables Linked to Climate Change Has a Stronger Effect on Aboveground Net Primary Productivity Than Does Phenological Change in Alpine Grasslands
Source: Front Plant Sci. 2022 Jan 4;12:798633. doi: 10.3389/fpls.2021.798633 (PMC8763838; doi:10.3389/fpls.2021.798633)
Supplement: Supplementary file 1 [file Data_Sheet_1.doc]

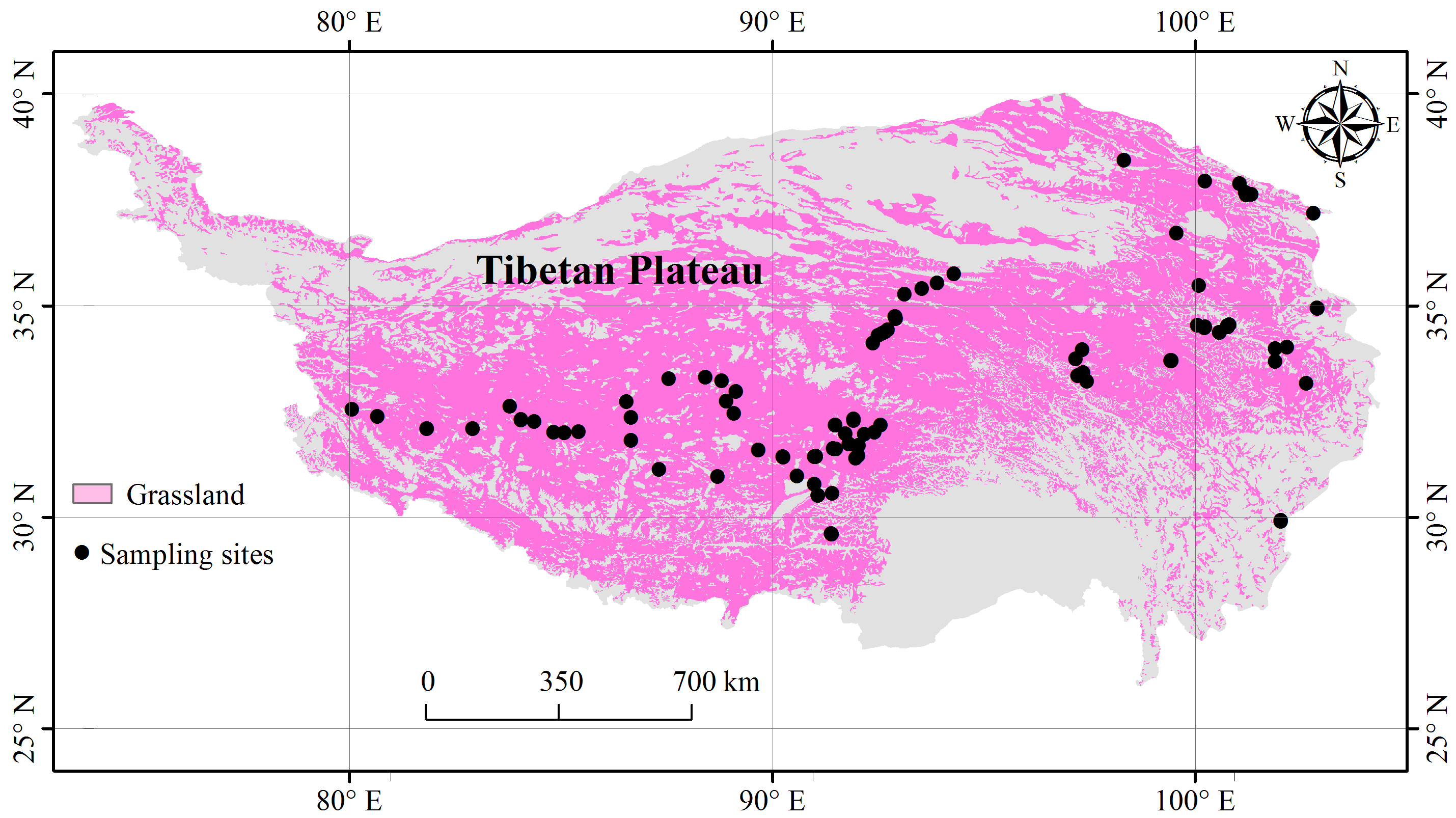


**Figure S1.** Location of aboveground biomass sampling sites on the Tibetan Plateau


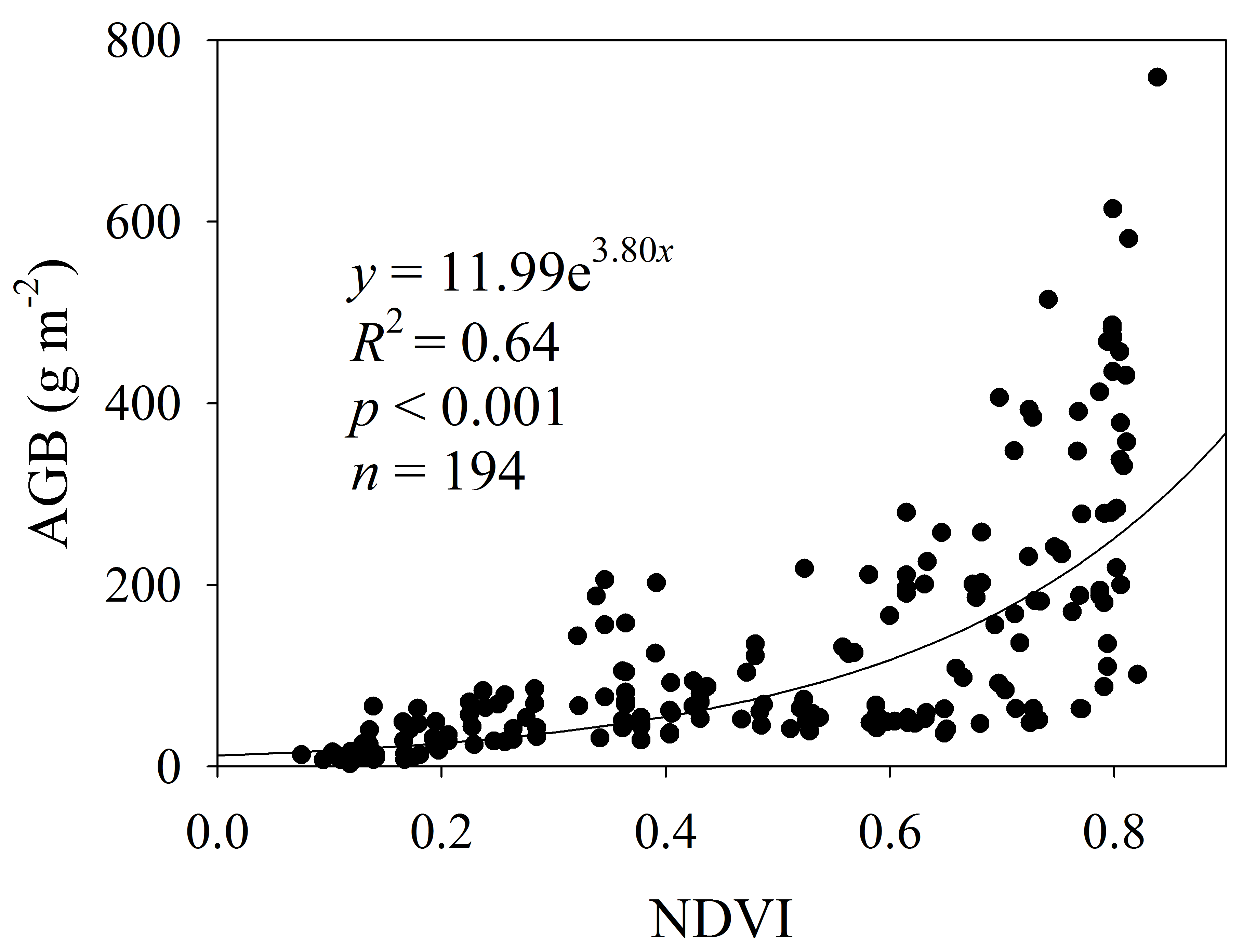


**Figure S2.** Relationships between aboveground biomass and normalized difference

vegetation index during July–August of 2000–2013 on the Tibetan Plateau

**Table S1** Linear relationships between growing-season precipitation (GSP), actual vapor pressure (E_a_), relative humidity (RH), minimum relative humidity (RH_min_), vapor pressure deficit (VPD), air temperature (T_a_), maximum air temperature (T_amax_), minimum air temperature (T_amin_), ≥5 ^o^C accumulated air temperature (AccT), the ratio of GSP to AccT (GSP/AccT), start of growing season (SOS), end of growing season (EOS), length of growing season (LOS), aboveground net primary production (ANPP) and year, showing the slopes.

| No | GSP | E_a_ | RH | RH_min_ | VPD | T_a_ | T_amax_ | T_amin_ | AccT | GSP/AccT | SOS | EOS | LOS | ANPP |
| --- | --- | --- | --- | --- | --- | --- | --- | --- | --- | --- | --- | --- | --- | --- |
| 56074 | 1.56 | 0.02 | -0.02 | -0.12 | 0.00 | 0.03 | 0.04 | 0.05 | 16.64 | 0.00 | -0.63 | 0.70 | 1.32 | 0.79 |
| 52633 | 3.12 | 0.01 | -0.03 | -0.28 | 0.00 | 0.02 | 0.02 | 0.05 | 3.66 | 0.00 | 0.13 | 0.24 | 0.11 | 1.10 |
| 52645 | 7.91 | 0.01 | -0.11 | -0.44 | 0.01 | 0.03 | 0.03 | 0.07 | 6.94 | 0.01 | -0.91 | -0.34 | 0.57 | 2.31 |
| 52657 | 5.55 | -0.06 | -0.41 | -0.47 | 0.04 | -0.03 | -0.02 | 0.02 | 21.23 | 0.00 | -1.36 | 0.64 | 2.00 | 1.37 |
| 52707 | 1.72 | -0.03 | -0.04 | -0.19 | -0.04 | -0.07 | -0.09 | -0.05 | 38.18 | 0.00 | -2.53 | 0.57 | 3.10 | 0.60 |
| 52754 | 3.24 | -0.03 | -0.25 | -0.47 | 0.02 | -0.02 | -0.01 | 0.00 | 15.82 | 0.00 | -1.27 | 0.98 | 2.25 | -4.91 |
| 52818 | 0.90 | 0.00 | -0.05 | -0.12 | 0.03 | 0.03 | -0.01 | 0.09 | 2.99 | 0.00 | -0.14 | -0.34 | -0.20 | 0.36 |
| 52908 | 7.46 | -0.02 | -0.06 | -0.13 | 0.00 | -0.06 | -0.09 | -0.01 | 5.90 | 0.02 | -1.07 | 1.55 | 2.61 | 0.18 |
| 52943 | 4.20 | 0.02 | -0.05 | -0.47 | 0.02 | 0.06 | 0.06 | 0.06 | -5.28 | 0.00 | 0.37 | -0.79 | -1.16 | -0.37 |
| 52974 | 6.94 | -0.04 | -0.32 | -0.45 | 0.05 | 0.02 | 0.04 | 0.06 | 15.66 | 0.00 | -0.50 | 0.35 | 0.85 | 0.11 |
| 56004 | 3.76 | 0.00 | -0.15 | -0.28 | 0.02 | 0.03 | 0.03 | 0.04 | 11.92 | 0.00 | -0.97 | -0.83 | 0.14 | 0.01 |
| 56018 | 3.59 | -0.04 | -0.45 | -0.64 | 0.06 | 0.02 | 0.05 | 0.01 | 11.96 | 0.00 | -0.12 | 1.15 | 1.26 | 1.26 |
| 56021 | 12.69 | -0.03 | -0.42 | -0.60 | 0.05 | 0.03 | 0.02 | 0.07 | 16.08 | 0.01 | -1.94 | -0.20 | 1.74 | 4.38 |
| 56033 | 7.34 | -0.02 | -0.38 | -0.47 | 0.05 | 0.04 | 0.03 | 0.07 | 9.25 | 0.01 | -1.70 | -0.67 | 1.02 | 0.90 |
| 56034 | 12.91 | 0.01 | -0.23 | -0.48 | 0.03 | 0.06 | 0.06 | 0.09 | 14.25 | 0.01 | -0.97 | -0.20 | 0.77 | 2.25 |
| 56043 | 8.15 | 0.03 | -0.22 | -0.42 | 0.05 | 0.10 | 0.07 | 0.15 | 9.49 | 0.00 | 0.38 | -0.40 | -0.78 | 3.65 |
| 56046 | 9.03 | -0.03 | -0.45 | -0.60 | 0.06 | 0.04 | 0.05 | 0.06 | 12.92 | 0.00 | -0.85 | 0.22 | 1.07 | 3.76 |
| 56065 | 13.27 | 0.01 | -0.13 | -0.35 | 0.02 | 0.03 | 0.01 | 0.11 | 14.07 | 0.01 | -0.81 | 0.81 | 1.62 | -1.44 |
| 56067 | 5.53 | 0.02 | -0.16 | -0.53 | 0.03 | 0.07 | 0.08 | 0.10 | 8.14 | 0.00 | -0.43 | -0.84 | -0.40 | -1.27 |
| 56151 | 6.06 | -0.02 | -0.37 | -0.52 | 0.06 | 0.05 | 0.07 | 0.05 | 8.54 | 0.00 | -0.60 | -0.41 | 0.19 | -9.37 |
| 55248 | 1.65 | -0.02 | -0.33 | -0.52 | 0.06 | 0.05 | 0.04 | 0.07 | 3.78 | 0.00 | 0.15 | 0.03 | -0.12 | -0.03 |
| 55279 | 4.47 | -0.05 | -0.49 | -0.96 | 0.06 | 0.02 | 0.05 | 0.02 | 4.65 | 0.00 | -0.05 | 0.42 | 0.47 | 1.25 |
| 55294 | -0.14 | 0.00 | -0.26 | -0.94 | 0.04 | 0.07 | 0.10 | 0.07 | 2.02 | 0.00 | -1.59 | -1.83 | -0.24 | -0.21 |
| 55299 | -4.74 | -0.08 | -0.60 | -1.18 | 0.06 | -0.01 | 0.06 | -0.03 | 4.31 | -0.01 | 0.61 | 1.38 | 0.77 | -10.48 |
| 55472 | -1.14 | -0.07 | -0.71 | -1.13 | 0.09 | 0.05 | 0.09 | 0.03 | 2.60 | 0.00 | 0.88 | 0.32 | -0.55 | 2.25 |
| 55493 | -15.23 | -0.09 | -1.00 | -1.98 | 0.13 | 0.10 | 0.18 | 0.04 | 8.81 | -0.01 | 0.83 | 0.18 | -0.65 | -5.93 |
| 55664 | 2.85 | -0.11 | -0.55 | -1.20 | 0.03 | -0.04 | -0.01 | -0.08 | 17.48 | 0.00 | 0.29 | 1.41 | 1.11 | 0.20 |
| 55773 | 1.29 | 0.02 | -0.14 | -0.50 | 0.02 | 0.05 | 0.03 | 0.09 | -5.60 | 0.00 | 1.54 | -0.69 | -2.23 | -0.11 |
| 56342 | -8.01 | -0.07 | -0.74 | -1.00 | 0.11 | 0.05 | 0.09 | 0.05 | 5.16 | -0.01 | -0.07 | -0.07 | 0.00 | -1.83 |
| 56038 | 5.97 | 0.01 | -0.35 | -0.54 | 0.06 | 0.11 | 0.12 | 0.14 | 12.72 | 0.00 | 0.10 | -0.32 | -0.42 | -1.15 |
| 56079 | 11.00 | 0.03 | -0.19 | -0.44 | 0.04 | 0.09 | 0.08 | 0.15 | 21.40 | 0.00 | -1.18 | -0.32 | 0.86 | 8.62 |
| 56152 | 3.72 | 0.04 | 0.07 | -0.28 | 0.00 | 0.05 | 0.09 | 0.03 | 9.34 | 0.00 | -0.12 | 0.19 | 0.31 | 1.87 |
| 56167 | 4.05 | -0.12 | -0.55 | -1.01 | 0.07 | -0.04 | 0.00 | -0.04 | 45.48 | 0.00 | -2.26 | 1.62 | 3.89 | -4.06 |
| 56173 | 9.78 | 0.04 | -0.16 | -0.23 | 0.04 | 0.09 | 0.11 | 0.11 | 13.26 | 0.00 | -0.73 | -0.79 | -0.06 | 2.86 |
| 56257 | -12.75 | -0.02 | -0.37 | -0.67 | 0.05 | 0.05 | 0.10 | 0.03 | 9.43 | -0.01 | 1.34 | 1.11 | -0.23 | -3.01 |
| 56357 | -9.48 | -0.05 | -0.53 | -0.92 | 0.08 | 0.05 | 0.12 | -0.01 | 28.75 | -0.01 | -1.08 | 0.79 | 1.87 | 0.13 |
